# Application of nano‐graphene oxide as nontoxic disinfectant against alpha and betacoronaviruses

**DOI:** 10.1002/vms3.584

**Published:** 2021-07-27

**Authors:** Hee‐Chun Chung, Van Giap Nguyen, Cheong Ung Kim, Hai‐Quynh Do, Bong Kyun Park, Yong Ho Park, Dae‐Sub Song, Aeri Kong, Jae‐Chul Ryu, Kyung‐Sun Kang

**Affiliations:** ^1^ Department of Veterinary Medicine Virology Lab College of Veterinary Medicine and Research Institute for Veterinary Science Seoul National University GwanAk‐Gu Seoul Korea; ^2^ Faculty of Veterinary Medicine Department of Veterinary Microbiology and Infectious Diseases Vietnam National University of Agriculture Hanoi Vietnam; ^3^ Department of Veterinary Microbiology College of Veterinary Medicine and Research Institute for Veterinary Science Seoul National University Seoul Republic of Korea; ^4^ Institute of Genome Research Vietnam Academy of Science and Technology Hanoi Vietnam; ^5^ Department of Pharmacy College of Pharmacy Korea University Sejong Republic of Korea; ^6^ Department of Medical Science University of California Los Angeles California USA; ^7^ Adult Stem Cell Research Center and Research Institute for Veterinary Science College of Veterinary Medicine Seoul National University Seoul Republic of Korea

**Keywords:** antiviral activity, coronaviruses, nano‐graphene oxide

## Abstract

New viruses are continuously emerging and recently there have been many great concerns on severe acute respiratory syndrome coronavirus (SARS‐CoV‐2). Nanographene oxide (nanoGO) has received much attention and is widely investigated to be utilised in therapy for infectious diseases by viruses. Thus, antiviral activity of nanoGO was evaluated using the porcine epidemic diarrhoea virus (PEDV), bovine coronavirus (BCoV), and SARS‐CoV‐2, which are all *Alpha‐* and *Beta‐coronavirus*. In a virus inhibition assay, the three viruses were inhibited by nanoGO in a dose‐dependent manner, including attempts in the presence of high serum solution which partially mimicked biological fluid.

## Graphical abstract

Our results indicated that nanoGO material revealed different levels of antiviral activity against different viruses.

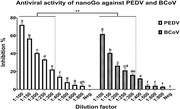

## 1 INTRODUCTION

Vaccines are widely accepted as an effective tool for responding to infectious diseases caused by viruses. However, they have limited effects against certain viruses, and the process for vaccine development usually begins after a new virus emerges and takes a long time to be commercialised. Recently, the coronavirus disease (COVID‐19) caused by SARS‐CoV‐2, a novel beta‐coronavirus originating from bats, has been inducing a global pandemic situation and severely threatening public health and global economy (Zhou et al., 2020). Several species belonging to *Alphacoronavirus* and *Betacoronavirus*, like PEDV or BCoV, have also been causing serious economic losses in livestock production (Luo et al., 2020). Thus, novel antiviral substances to broadly and effectively control virus infections are being largely demanded.

Up to date, there are several kinds of agents that are known to have antiviral activities (Chen & Liang, 2020). Graphene oxide (GO), an ultra‐thin carbon material, is one of these agents that has been demonstrated with the function of broad‐spectrum antiviral activity (Song et al., 2015; Ye et al., 2015). GO has also been regarded as an excellent candidate for anti‐inflammatory and ‐microbial therapy (Lee et al., 2020).

NanoGO, a graphene oxide with nanoscale lateral dimension (Sanchez et al., 2012), displays antiviral activity which was demonstrated in previous studies. In brief, GO in combination with silver nanoparticles can inhibit the infectivity of FeCoV, IBDV and PRRSV (Chen et al., 2016; Du et al., 2018). Additionally, label‐free GO possibly captures and changes the structure of surface protein of Enterovirus A71 and avian influenza virus serotype H9N2 (Song et al., 2015). Iannazzo et al. (2018) constructed graphene quantum dots (GQDs) based systems that highly inhibited HIV replication in vitro. The biological characterisations of nanoGO vary based on its own physical properties like size, oxidative level, as well as those of its additional groups. However, the biological properties of nanoGO might be affected in the presence of serum (Song et al., 2020) and the cytotoxic effects of GO is size‐dependent (Zhao et al., 2016), thus its application in therapy has been limited so far. Faced with the serious pandemic of COVID‐19, nanoGO and its deliveries were considered as a great virucidal material to be applied in antiviral surfaces and coatings (Palmieri & Papi, 2020; Seifi & Reza Kamali, 2021; Srivastava et al., 2020). Based on the great potential antiviral activity of this substance mentioned above, this study was carried out to test the antiviral properties of GO against coronaviruses (PEDV, BCoV and SARS‐CoV‐2) in the presence of high organic materials (5% FBS).

## 2 MATERIALS AND METHODS

In this study, nanoGO was evaluated for antiviral activity in a solution partially mimicking biological fluid with the use of serum. The active ingredient, 1% nanoGO solution (3 mg/mL), was prepared according to the previous publication (Lee et al., 2020). The morphology of nanoGO was observed by FE‐SEM: XL30 (Philips), HR‐TEM: JEM‐ARM200F (Cold FEG, JEOL Ltd, Japan) and AFM: SPM‐9700HT (Shimadzu). The size of particles was analysed by CPS DC24000 particle analyser (CPS instrument, USA). Other characteristics of nanoGO were identify by Raman Spectroscopy NRS‐3300 (Japan), FT‐IR (TENSOR27, Bruker, Germany), XRD (SmartLab, Rigaku, Japan) and XPS (AXIS SUPRA, Kratos, UK)

Steps in testing the antiviral activity of nanoGO are summarised as follows. The original solution of nanoGO was diluted in DMEM supplemented with 5% FBS. Each dilution was mixed with an equal volume of virus solution with known titre or with suitable cell culture medium for control of nanoGO toxicity. The mixtures of virus‐nanoGO and nanoGO control were incubated at a defined temperature for 60 minutes. Subsequently, viral titrations of each mixture were performed on a susceptible cell line.

More specifically, for antiviral activity against PEDV/ BCoV, nanoGO was diluted 50‐ to 800‐fold in DMEM supplemented with 5% FBS. Each dilution was mixed with equal volume of either PEDV (DR13 strain) or BCoV (BC94 strain) having a titre of 10^7^ TCID_50_/mL. The incubation time at room temperature was 60 minutes. PEDV and BCoV after treatment with nanoGO were titrated on Vero cells using the methods described previously (Hansa et al., 2013; Song et al., 2003). A maximum dilution factor, in which the virus titre was reduced by at least 4 log10, was determined to be an effective dilution factor (Agriculture‐Forestry and Livestock Quarantine Headquarters, 2018). Antiviral effect of nanoGO was expressed by % inhibition, which was calculated as follows: [log_10_ (TCID_50_/mL of virus) – log_10_ (TCID_50_/mL of treatment)]/ (log_10_ (TCID_50_/mL of virus) × 100% (Chen et al., 2016). Additionally, immunofluorescence assays (IFA) were performed to detect the replication of living virus post‐treatment more precisely. An IFA was performed 24 hours post‐inoculation using the PEDV IFA kit (MEDIAN Diagnostics, South Korea) and BCoV 1st antibody (provided by MEDIAN Diagnositic). Statistical analysis was performed using GraphPad Prism version 8.0.2.

For antiviral activities against SARS‐CoV‐2, the neutralising test was conducted using the previous method (Manenti et al., 2020) with modifications. The nanoGO solution was serially diluted two‐fold in DMEM supplemented with 5% FBS. Subsequently, the SARS‐CoV‐2 (BetaCoV/Korea/KCDC03/2020) of 25TCID_50_/mL was mixed with equal volume of the diluted nanoGO. The mixtures were incubated for 60 min at 37°C. After incubation, 0.1 mL of each nanoGO mixture was infected to a monolayer of Vero E6 cells. The presence/absence of cytopathic effect (CPE) was monitored daily for 5 days. The neutralising titres were expressed as the reciprocal of the highest dilution, which resulted in the inhibition of CPE. All experiments related to SARS‐CoV‐2 were performed in BL3 facility.

## 3 RESULTS

In this study, using improved Hummer's method described before, we obtained the nanoGO material sharing similar size with that of the study conducted by Lee et al. (2020). In brief, FE‐SEM results indicated that the lateral size of nanoGO particles was less than 50 nm with irregular shapes. Particle analysis results indicated that most of the material were less than 30 nm in size with the average size of 20 nm (Figure S1a). HR‐TEM had previously been applied to observe the layer structure of nano‐particles (Çelik et al., 2017; Gonçalves et al., 2014; Yang et al., 2014). Therefore, we applied this method in combination with image analysis to determine the diameter of particles. The results revealed that most of nanoGO particles contained 1 to 3 layers (Figure S1b). AFM results also indicated that the height of nanoGO particles were around 1–2 nm (Figure S1c), supporting the HR‐TEM result. Raman spectra analysis exhibited the D peak of approximately 1350 cm^−1^ and a G band at 1600 cm^−1^, which are known peaks specific to GO (Figure S1d). Functional groups and oxidative state of nanoGO were measured by the Fourier‐transform infrared spectroscopy (FT‐IR), X‐ray photoelectron spectroscopy (XPS) and X‐ray diffraction (XRD). GQD's FT‐IR spectrum analysis revealed the major peaks of O–H (around 3420 cm^−1^), C–H (2928 and 2850 cm^−1^), COOH (1730 cm^−1^), C = O (1630 cm^−1^), CH_2_ (1465 cm^−1^) and C–O (1044 cm^−1^) (Figure S2a). XRD analysis clearly showed a peak at a low diffraction angle (2*θ* = 10.32° with an interlayer spacing about 8.57 A°) which represents a high oxidative level of this material (Figure S2b). In XPS analysis, the binding energy of C–C (284.50 eV), C–O (286.68 eV) and C = O (288.36 eV) were measured (Figure S2c).

The toxicological effect is the highest criteria for consideration before applying nanomaterial in reality. In this study, cytotoxicity of nanoGO, which was represented as the presence of CPE, was not observed in Vero cell at the lowest dilution of 1/50 (Table S2). Additionally, CPE was not observed at the dilution factor of ½ when we performed neutralisation test against SARS‐CoV‐2 (Figure 3). Therefore, it is reasonable to conclude that there was no cytotoxicity of nanoGO at the investigated concentration.

**FIGURE 1:**
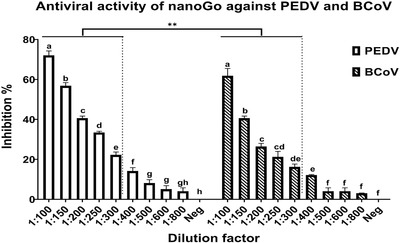
Antiviral activity of nanoGO against PEDV and BCoV in different dilution factors. The denoted letters indicate the statistically significant differences among dilution factors in each group (*p* < 0.05). The asterisk demonstrated the significant differences among groups (*p < 0.05; **p < 0.01)

**FIGURE 2:**
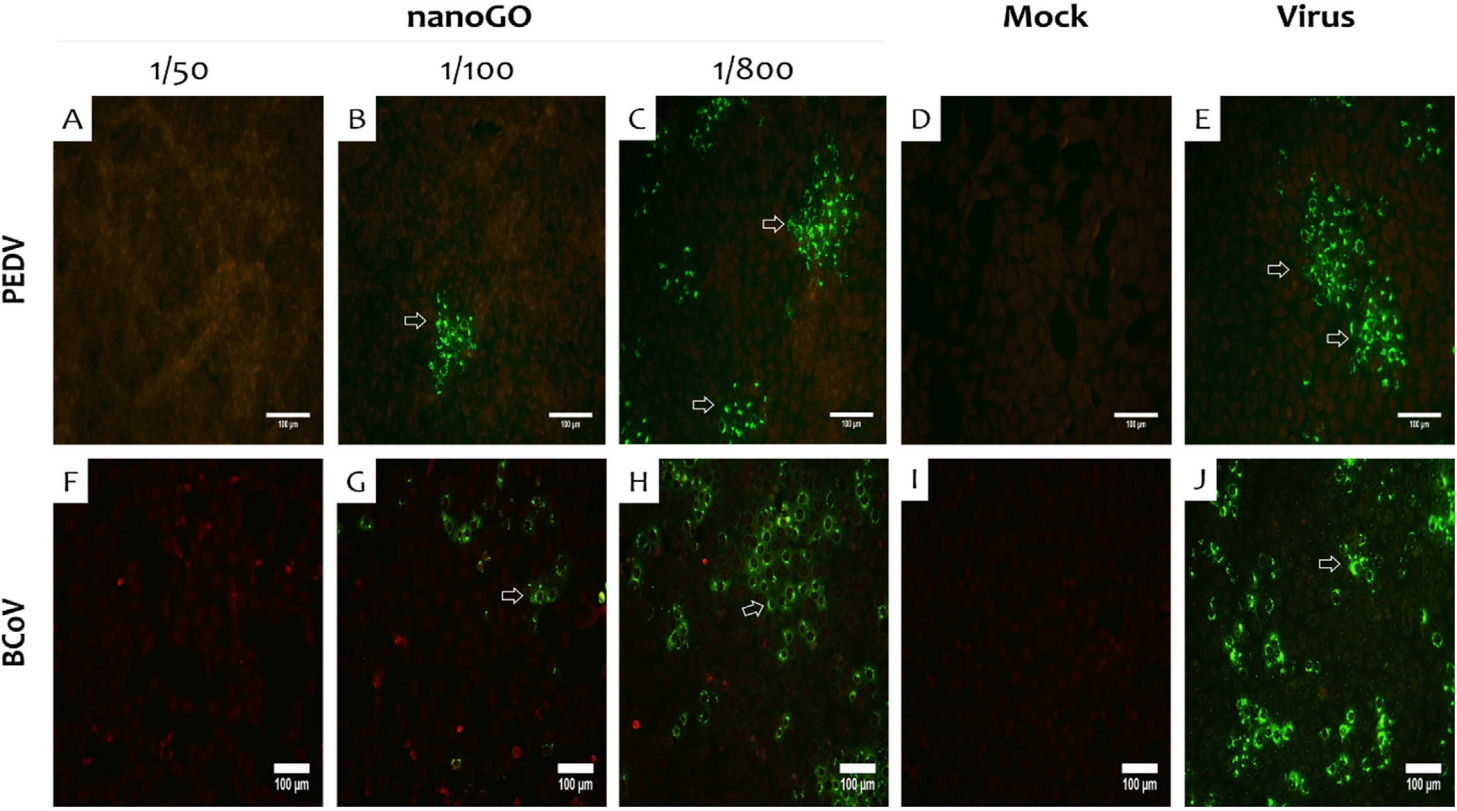
IFA assay demonstrating the replication of PEDV and BCoV post nanoGO incubation. Cells with fluorescent signals (arrows) were virally infected. The higher number of fluorescent cells, the higher the amount of viral replication

The antiviral activity of nanoGO was initially demonstrated for coronaviruses (PEDV, BCoV) inducing diseases of animals. It was observed that increasing the dilution of nanoGO (1/50 to 1/800) increased the titres of PEDV/ BCoV from 0.0 to 6.3/6.4 log_10_ TCID_50_, gradually approaching the titres in the mock‐treated groups (both were 6.6 log_10_ TCID_50_). The results implied that nanoGO exerted in vitro antivirus activity against PEDV/ BCoV in a dose‐dependent manner. In detail, the highest antiviral activities of nanoGO against PEDV and BCoV were achieved at 72.1% and 61.9%, respectively. However, there was little to no antiviral effect of nanoGO obtained for PEDV and BCoV when the nanoGO solution was dissolved to the concentrations of 0.00125% and 0.2% (*p* > 0.05) (Figure 1). Furthermore, at up to 1/300 dilution, nanoGO revealed the more effective antiviral agent against PEDV than against BCoV (*p* < 0.01) (Figure 1). At 100 times diluted, nanoGO blocked more efficiently the replication of viruses (columns 2 and 3, Table S1). The virucidal activity of nanoGO was also confirmed by IFA staining (Figure 2). The infected cells (green fluorescence) were not observed at low dilution (1/50) of nanoGO (Figure 2a, f). However, the active agent at a dilution of 1/100 or higher (Figure 2b, c, g and h) was unable to completely inactivate the viruses.

**FIGURE 3:**
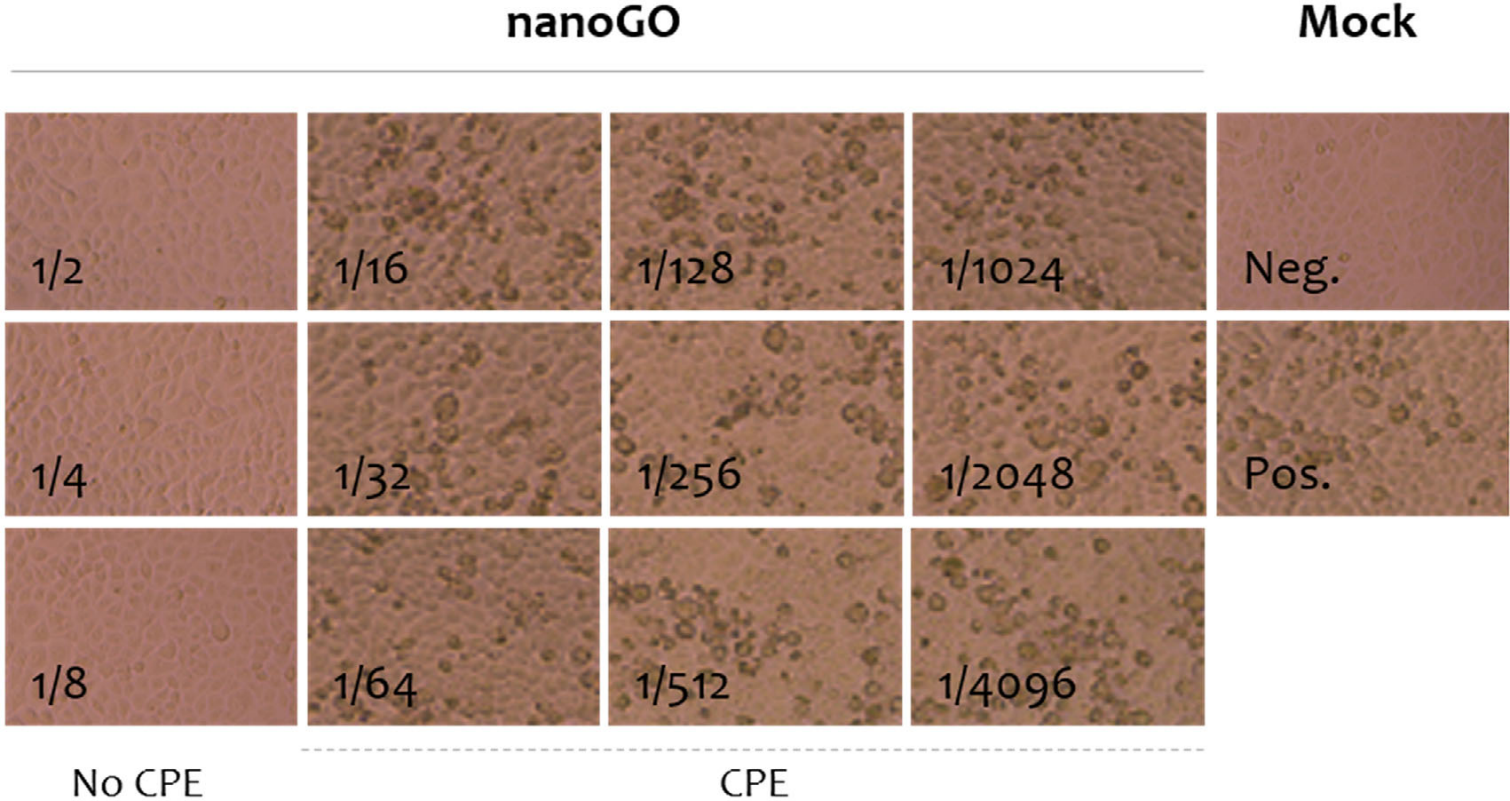
The cytopathic effects (CPE) induced by SARS‐CoV‐2 under different concentrations of nanoGO. It was observed that nanoGO at a dilution higher than 1/8 failed to completely inhibit the replication of the virus (CPE positive)

The antiviral activity of nanoGO was also detected for another coronavirus, SARS‐CoV‐2 which is the causative agent of the COVID‐19 pandemic (Zhou et al., 2020). As shown in Figure 3, nanoGO in the range of 1/2–1/8 dilution inhibited the replication of SARS‐CoV‐2 (no cytopathic effects were observed). From the 1/16 dilution, nanoGO failed to inactivate the replication of the virus. However, the level of SARS‐CoV‐2 inhibition was not determined in this study. Combining the results presented in Table S1 and Figures 1, 2, 3, it was inferred that nanoGO was a broad‐spectrum antiviral agent against different coronaviruses causing diseases in animals and humans.

## 4 DISCUSSION

In literature, graphene oxide (GO) is known to be a biocompatible substance with no indication for causing any harmful effects in experimental animals (Lee et al., 2020) and with low cytotoxicity to cell lines (Kuo et al., 2017; Sametband et al., 2014; Ye et al., 2015). Previous studies indicated that the bactericidal and cytotoxic activities of nanoGO depended on its size (Liu et al., 2012; Zhao et al., 2016). In brief, nanoGO with lateral dimensions larger than 50 nm significantly reduced the viability of *Escherichia coli* and macrophage cells. In this study, we used nanoGO with the average size of 20 nm, which caused no harmful effects on Vero cells. Additionally, this type of nanoGO was also demonstrated as safe in in vivo models (Lee et al., 2020).

NanoGO is known for its inhibition property against a wide range of viruses, both non‐enveloped and enveloped (Chen et al., 2016), DNA and RNA viruses (Ye et al., 2015). NanoGO could trigger the cytokine response that might inhibit the viral replication process in the host cell (Lategan et al., 2018). The anti‐microbial effects of nanoGO highly depend on several factors like exposed time, concentration, and lateral size. Furthermore, virucidal activities of nanoGO are also varied against different viruses. NanoGO and its deliveries significantly inactivated PRV and PEDV at the concentration of 6 μg/mL after 1 hour by destroying viral morphology (Ye et al., 2015). Chen et al. (2016) applied graphene oxide alone or in combination with nano‐silver to inhibit different types of viruses. The results indicated that only GO–Ag showed the effective antiviral activities against low titre of FCoV and IBDV at 0.125 mg/mL while GO only inhibited the infection of FeCoV after a 1‐hour treatment. However, the authors using another method for preparing GO resulted in a difference of oxidised carbon material (Marcano et al., 2010). Antiviral activity of GO against *Alphacoronavirus* and *Betacoronavirus* was demonstrated in this study with an expansion to the emerging SARS‐CoV‐2 (Figures 1, 2, 3). Our results were highly supported by a recent study that confirmed the trapping effect of nanoGO against SARS‐CoV‐2 (Maio et al., 2020). However, our study also demonstrated other virucidal aspects of GO by finding that its viral inhibition remained to a certain extent in the presence of high organic material (5% FBS). This fact should be further investigated due to a significant difference in dose‐ response of nanoGO against PEDV/ BCoV (Figures 1 and 2) and SARS‐CoV‐2 (Figure 3).

Overall, this study demonstrated the antiviral activity of nanoGO in a setting that partially mimicked biological fluid. This results also suggest that the antiviral activity of nanoGO could be achieved without causing harm to the cell. The concentration dependent fashion of viral inhibition was observed for all enveloped viruses of PEDV, BCoV and SARS‐CoV‐2. However, since antiviral activity on non‐enveloped virus was not evaluated, further study is required.

## AUTHOR CONTRIBUTIONS

HCC, YHP, KSK, DSS, CUK and BKP conceptualisation of the study. HCC, HQD, and VGN analysed the data. HCC, DSS, JCR, AK and KSK did data curation and drew up the manuscript. HCC, YHP, AK and KSK reviewed the manuscript. All authors read and approved the final manuscript.

## CONFLICT OF INTEREST

The authors declare that there is no conflict of interest.

### PEER REVIEW

The peer review history for this article is available at https://publons.com/publon/10.1002/vms3.584.

## Supporting information


**TABLE S1**. Antiviral activity of nanoGO against PEDV and BCoV

**TABLE S2**. Cytotoxic analysis of nanoGO on Vero cell

**FIGURE S1**. Physical properties of nanoGO using in this study. (a) Representative FE‐SEM image of nanoGO and the diagram showing the size distribution of nanoGO particles. NanoGO particles were circled and provided with dimensions. (b) Representative HR‐TEM image showing the layered structure of nanoGO. The height of layers were determined. (c) Represent the AFM image and thickness analysis of two represent nanoGO particles. (d) Representative Raman spectra of nanoGO

Supporting Information

Supporting Information

Supporting Information

**FIGURE S2**. Characteristic analysis of nanoGO. (a) FT‐IR spectra of nanoGO. (b) XRD spectrum of nanoGO. (c) XPS spectrum of nanoGO, C1s

Supporting Information

Supporting Information
