# Supplementary material for: Application of nano‐graphene oxide as nontoxic disinfectant against alpha and betacoronaviruses
Source: Vet Med Sci. 2021 Jul 27;7(6):2434–9. doi: 10.1002/vms3.584 (PMC8604132; doi:10.1002/vms3.584)
Supplement: Supplementary file 1 — TABLE S1. Antiviral activity of nanoGO against PEDV and BCoV [file VMS3-7-2434-s004.docx]

**Supplement table 1.** Antiviral activity of nanoGO against PEDV and BCoV

| **Treatment**  **condition** | **NanoGO dilution** | **PEDV** | **BCoV** |
| --- | --- | --- | --- |
|  |  | ***Mean titer (log10 TCID_50_)*** | ***Mean titer (log10 TCID_50_)*** |
| Virus + nanoGO | 1/50 | 0.0 | 0.0 |
|  | 1/100 | 1.8 | 2.5 |
|  | 1/150 | 2.8 | 3.9 |
|  | 1/200 | 3.9 | 4.8 |
|  | 1/250 | 4.4 | 5.2 |
|  | 1/300 | 5.1 | 5.5 |
|  | 1/400 | 5.6 | 5.8 |
|  | 1/500 | 6.0 | 6.3 |
|  | 1/600 | 6.2 | 6.3 |
|  | 1/800 | 6.3 | 6.4 |
| Virus | NA^*^ | 6.6 | 6.6 |

^*^Not applicable (NA); ^**^ % inhibition = [log_10_ (TCID50/ml of virus) – log_10_ (TCID_50_/ml of treatment)]/ (log_10_ (TCID_50_/ml of virus) x 100%. Underlines denote a maximum dilution factor, in which the virus titer of the treatment group (1.8 for PEDV and 2.5 for BCoV) was reduced by at least 4 log10 in comparing to that of the mock- treated group (6.6 for both PEDV and BCoV).
