# Supplementary material for: Application of nano‐graphene oxide as nontoxic disinfectant against alpha and betacoronaviruses
Source: Vet Med Sci. 2021 Jul 27;7(6):2434–9. doi: 10.1002/vms3.584 (PMC8604132; doi:10.1002/vms3.584)
Supplement: Supplementary file 2 — TABLE S2. Cytotoxic analysis of nanoGO on Vero cell [file VMS3-7-2434-s003.docx]

**Supplementary Table 2.** Cytotoxic analysis of nanoGO on Vero cell

| NanoGO  Toxicity control | **NanoGO dilution** | **CPE test** |
| --- | --- | --- |
|  |  | ***Mean titer (log10 TCID_50_)*** |
|  | 1/50 | NC^*^ |
|  | 1/100 | NC |
|  | 1/150 | NC |
|  | 1/200 | NC |
|  | 1/250 | NC |
|  | 1/300 | NC |
|  | 1/400 | NC |
|  | 1/500 | NC |
|  | 1/600 | NC |
|  | 1/800 | NC |

^*^ NC; No cytopathic effect (NC < 0.5).
