# Supplementary figures and images for: Application of nano‐graphene oxide as nontoxic disinfectant against alpha and betacoronaviruses
Source: Vet Med Sci. 2021 Jul 27;7(6):2434–9. doi: 10.1002/vms3.584 (PMC8604132; doi:10.1002/vms3.584)

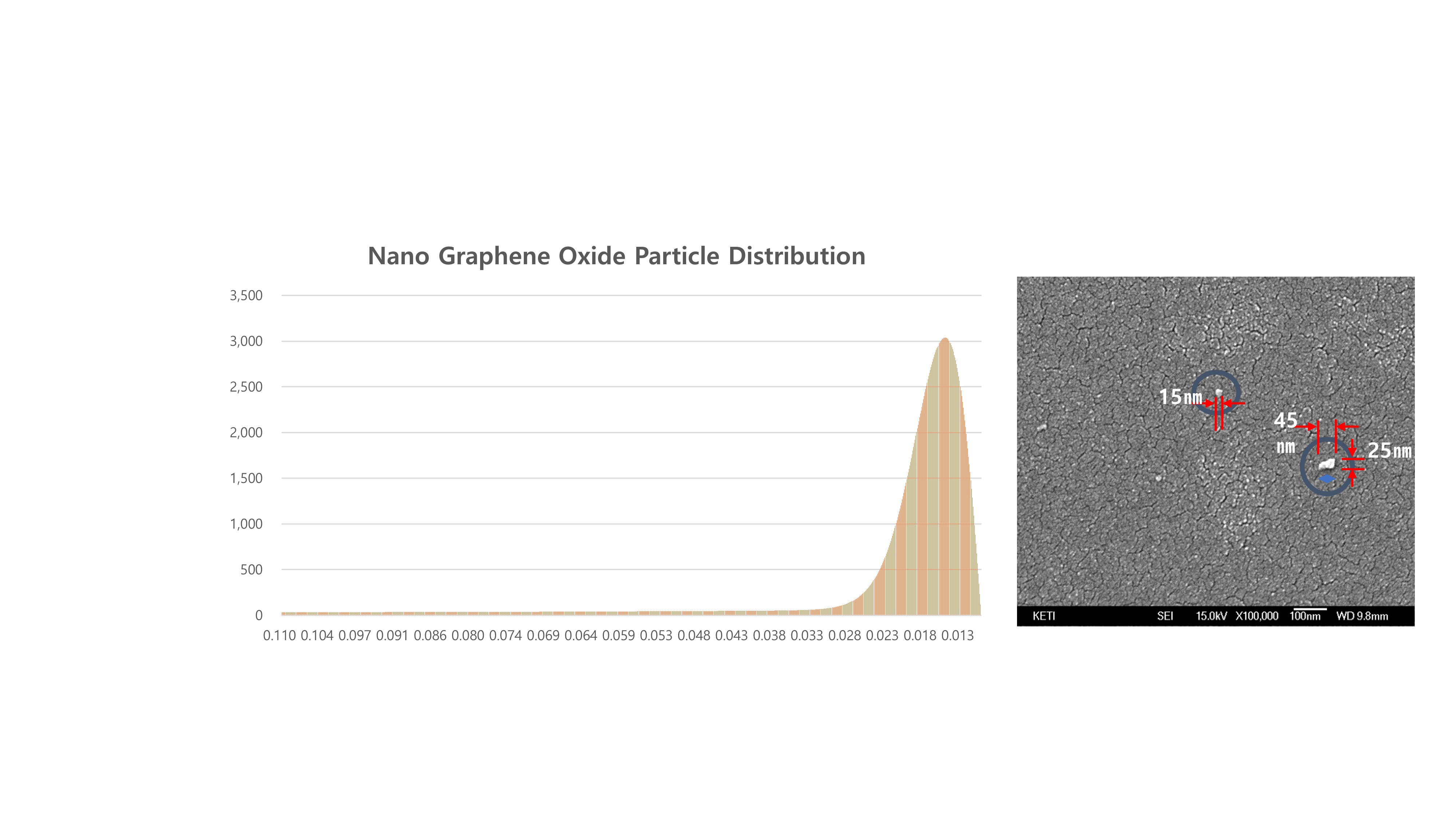

Supplement: Supplementary file 3 — FIGURE S1. Physical properties of nanoGO using in this study. (a) Representative FE‐SEM image of nanoGO and the diagram showing the size distribution of nanoGO particles. NanoGO particles were circled and provided with dimensions. (b) Representative HR‐TEM image showing the layered structure of nanoGO. The height of layers were determined. (c) Represent the AFM image and thickness analysis of two represent nanoGO particles. (d) Representative Raman spectra of nanoGO [file VMS3-7-2434-s005.jpg]

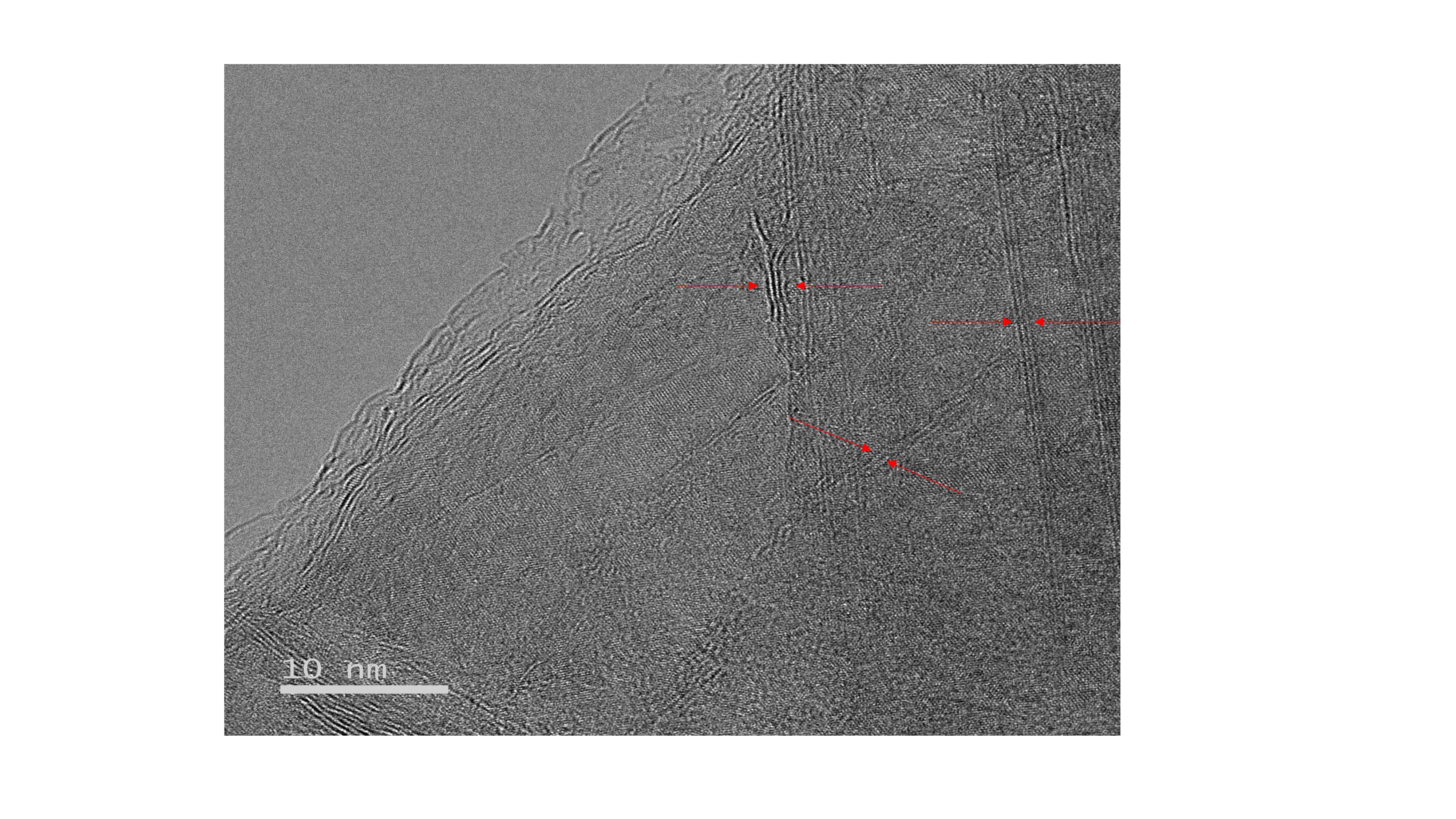

Supplement: Supplementary file 4 — Supporting Information [file VMS3-7-2434-s007.jpg]

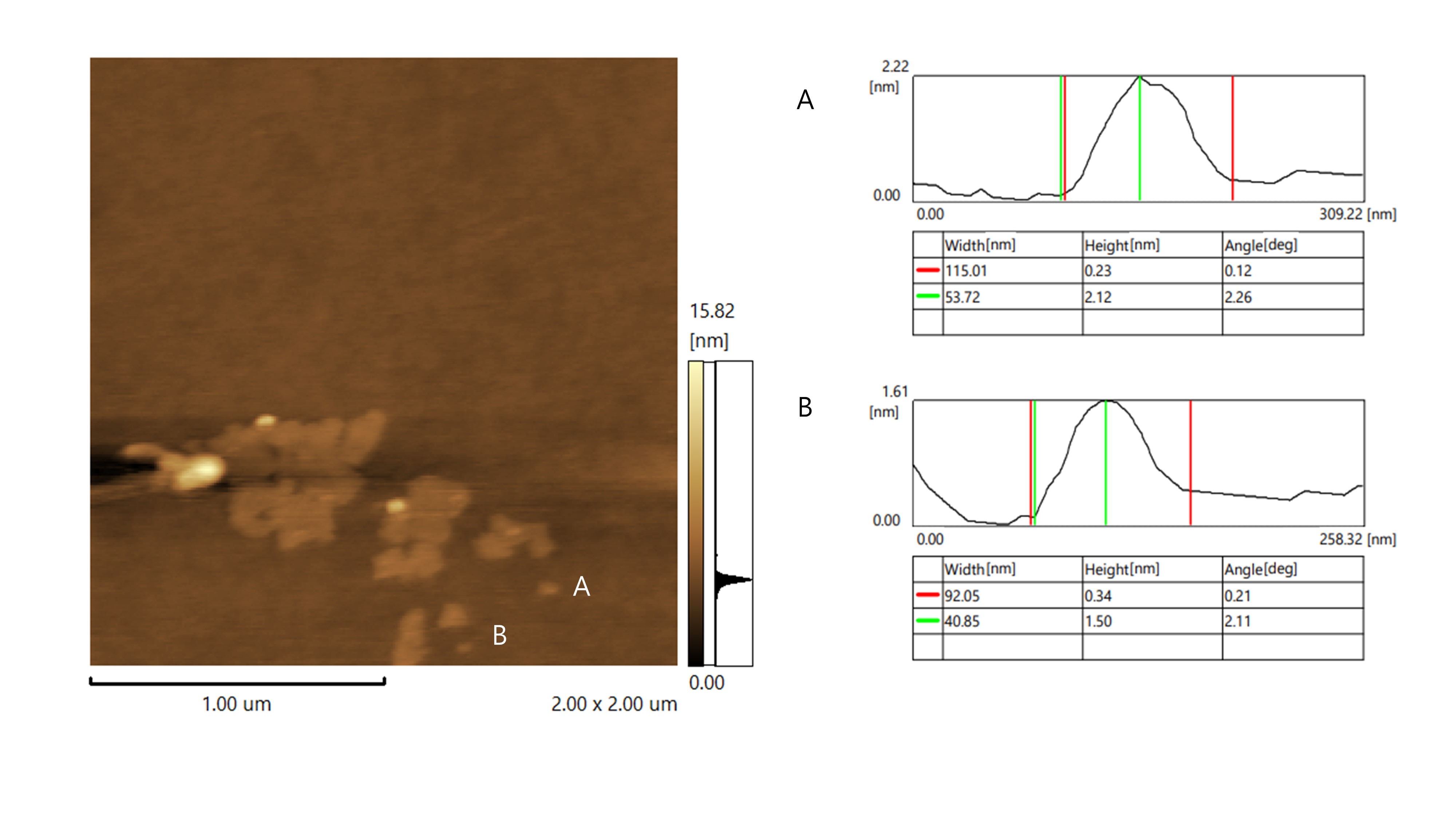

Supplement: Supplementary file 5 — Supporting Information [file VMS3-7-2434-s009.jpg]

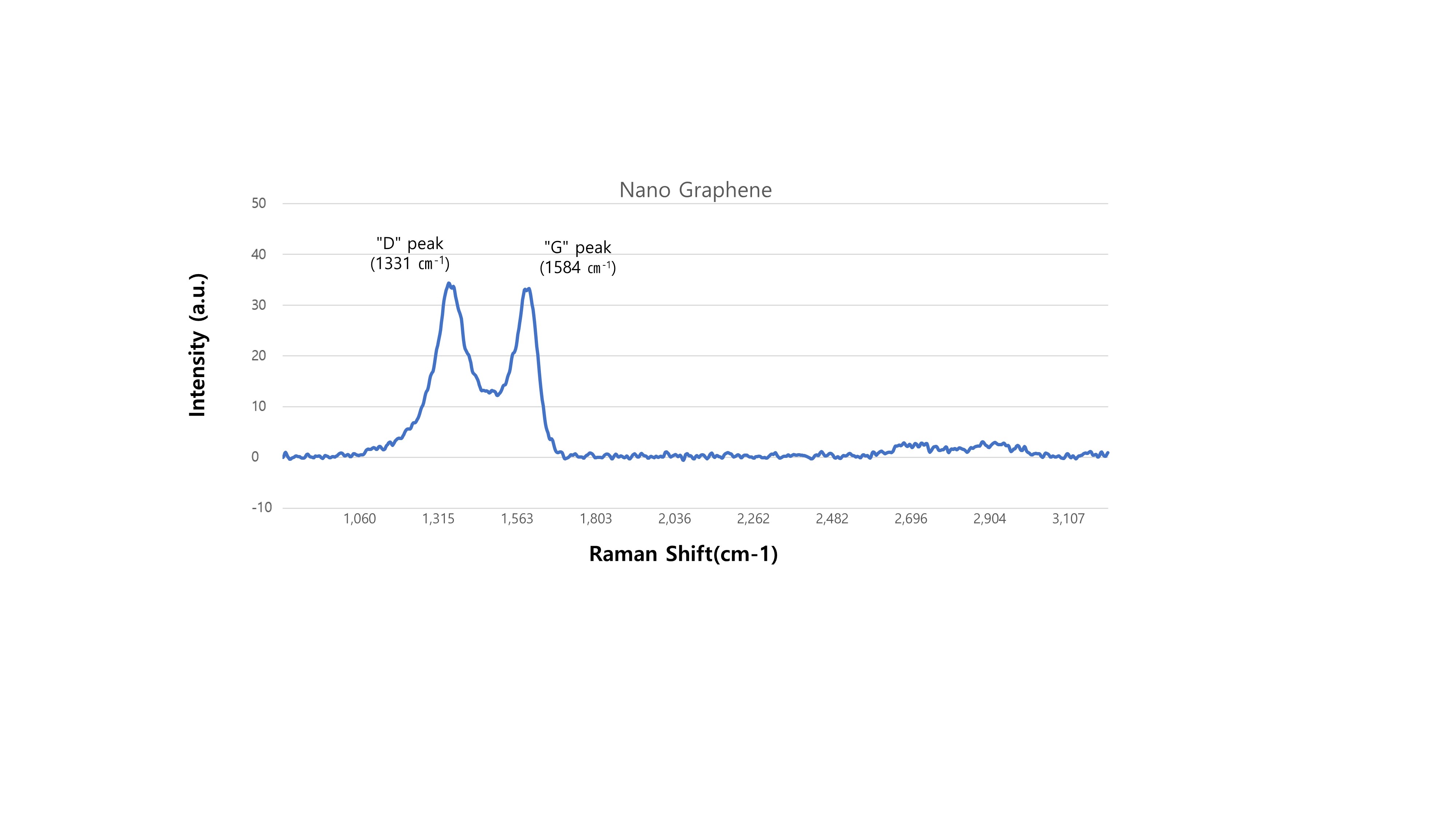

Supplement: Supplementary file 6 — Supporting Information [file VMS3-7-2434-s001.jpg]

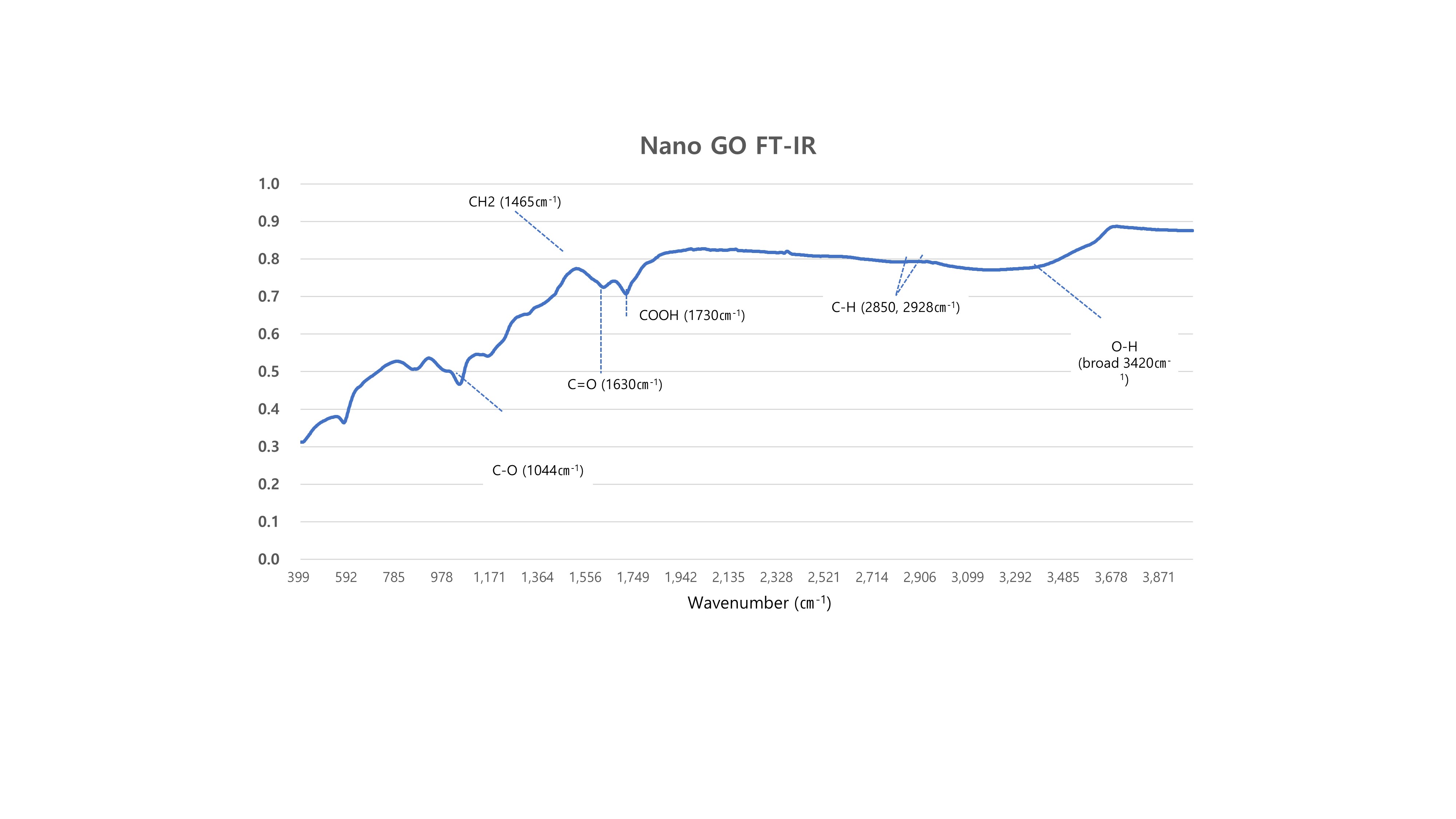

Supplement: Supplementary file 7 — FIGURE S2. Characteristic analysis of nanoGO. (a) FT‐IR spectra of nanoGO. (b) XRD spectrum of nanoGO. (c) XPS spectrum of nanoGO, C1s [file VMS3-7-2434-s006.jpg]

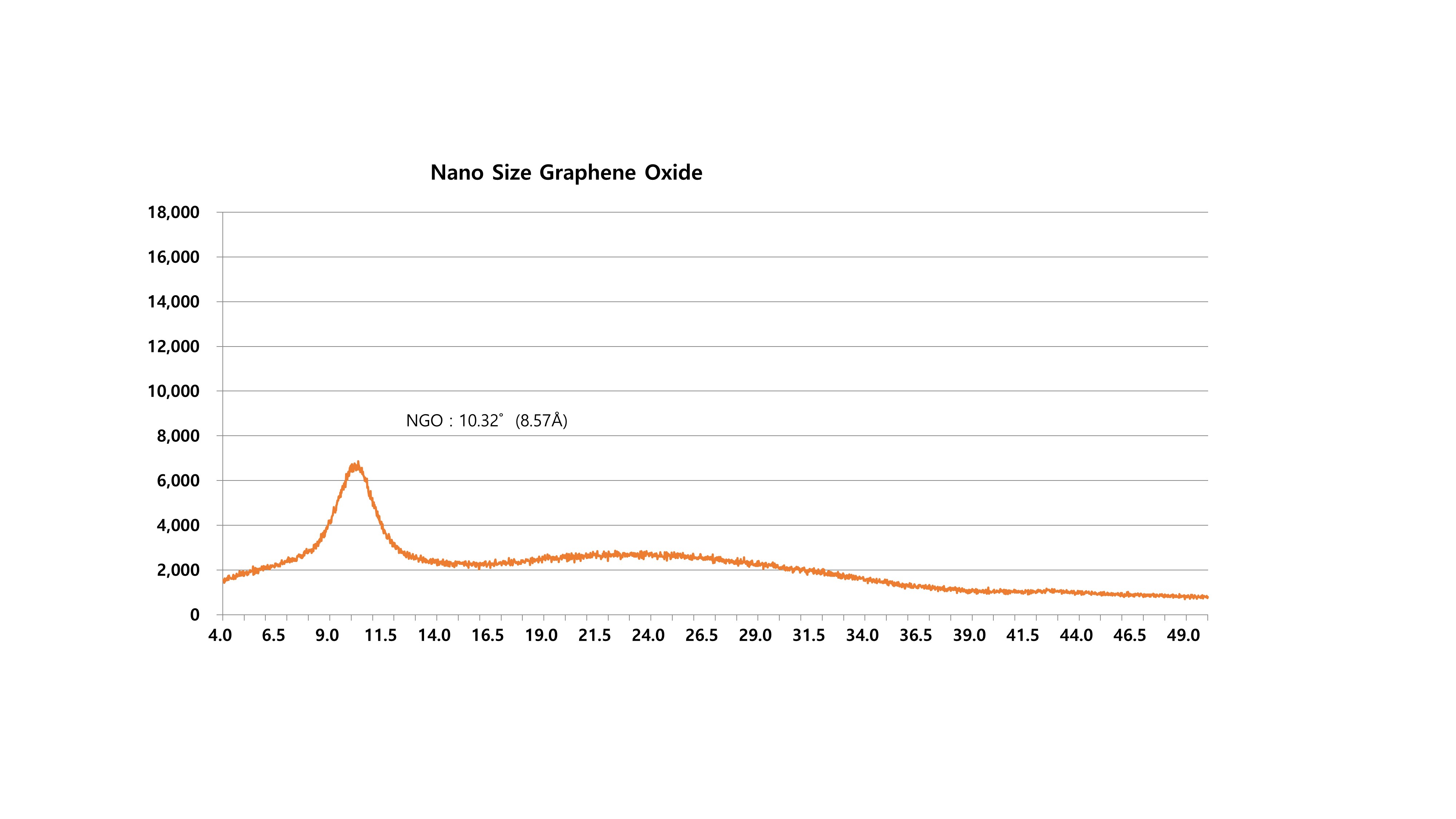

Supplement: Supplementary file 8 — Supporting Information [file VMS3-7-2434-s008.jpg]

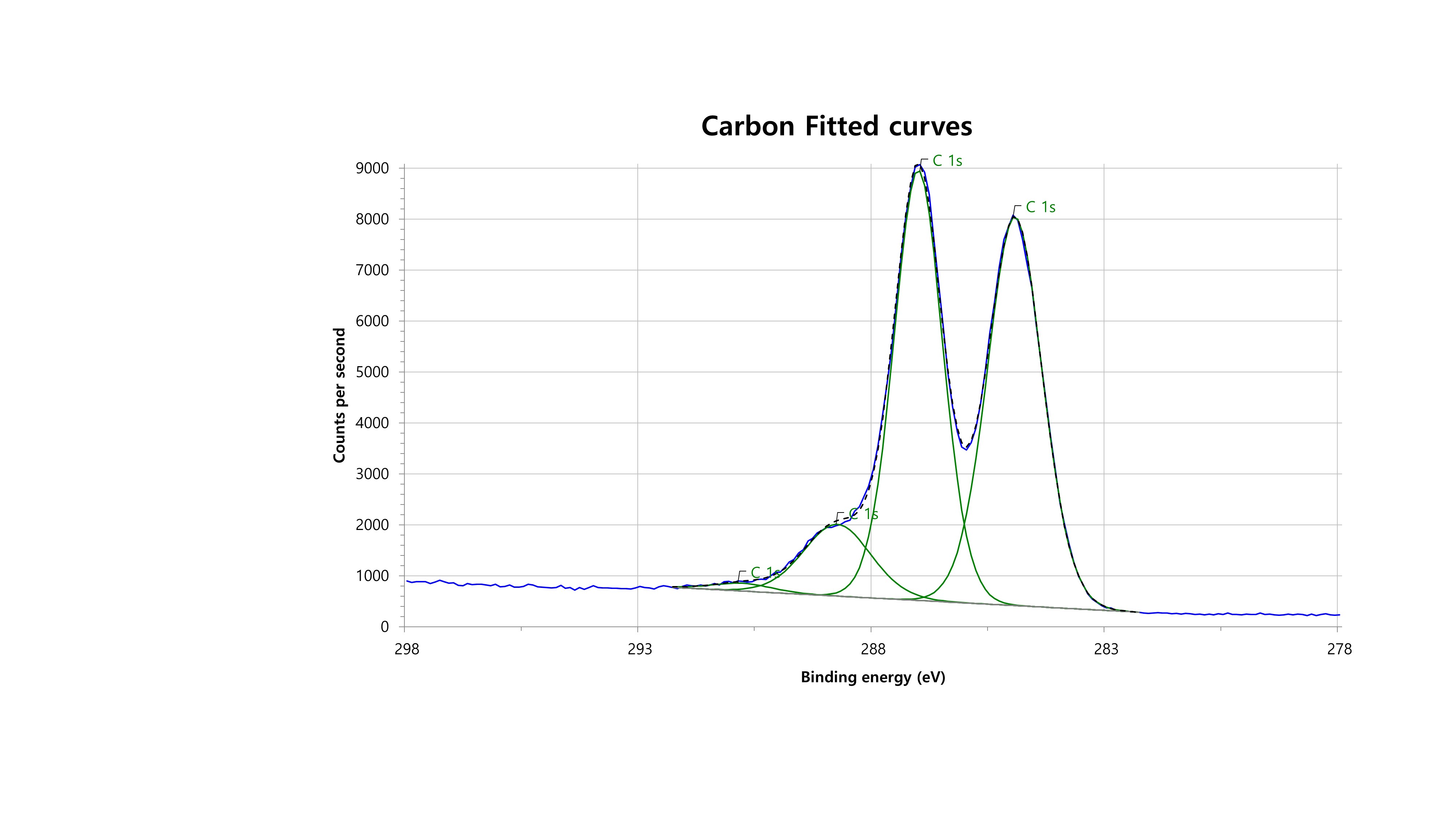

Supplement: Supplementary file 9 — Supporting Information [file VMS3-7-2434-s002.jpg]
